# Genotypic variation in garlic (*Allium sativum* L.) for salinity tolerance: role of antioxidant enzymes and bulb ionic ratios in yield maintenance

**DOI:** 10.3389/fpls.2026.1825262

**Published:** 2026-05-12

**Authors:** Satish Kumar Sanwal, Arvind Kumar, Antim Kundu, Hari Kesh, Ashwani Kumar

**Affiliations:** 1 Division of Crop Improvement, Indian Council of Agricultural Research (ICAR) Central Soil Salinity, Research Institute, Karnal, Haryana, India; 2 College of Horticulture and Forestry, Central Agricultural University (Imphal), Pasighat, Arunachal Pradesh, India

**Keywords:** garlic, nutrient imbalance, physiology, salt stress, tolerance mechanism

## Abstract

Salinity stress severely limits garlic (*Allium sativum* L.) yield by affecting plant growth, physiology, and bulb development. In field conditions, reproducing the same salinity stress each year is not possible, which restricts efficient parental selection and slows the pace of breeding salinity-tolerant genotypes. Thus, in the present study, 36 garlic genotypes were evaluated under control and salinity stress (EC_iw_ ≈ 7 dS m^-^¹) for morpho-physiological and biochemical traits. Based on yield reduction under salinity stress compared to control, six genotypes (G-304, Yamuna Safed-8, G-1, Yamuna Safed-3, G-324, and Yamuna Purple-10) were identified as salt-tolerant, maintaining the yield loss below 15%. Under salinity stress, Na^+^/K^+^ ratio and oxidative stress markers H_2_O_2_ and MDA increased in all genotypes; however, susceptible genotypes suffered severe membrane damage and higher yield losses. In contrast, tolerant genotypes showed higher antioxidant enzyme [catalase (CAT), ascorbate peroxidase (APX), superoxide dismutase (SOD), and peroxidase (POX)] activity than susceptible genotypes, which helped them mitigate salt stress. In the final step, stepwise regression was applied to determine the most reliable trait for screening salt tolerance in garlic genotypes. It was found that equatorial diameter was the primary determinant of bulb yield in garlic under stress as well as under control conditions, while ionic traits, specifically bulb Na^+^/K^+^, also contributed significantly to bulb yield under stress. The identified tolerant genotypes and key traits provide valuable resources for breeding salinity-tolerant garlic cultivars.

## 1 Introduction

Soil salinity is one of the major abiotic stresses that significantly reduce the growth and fruit yield of many vegetable crops like garlic and onion (Chaudhry and Sidhu, 2022). A total of 1,381 million ha (Mha), or 10.7% of the total global land area, featured a surplus of sodium, chloride, and sulfate ions (Food and Agriculture Organization of the United Nations, 2024). Soil salinization is a growing global concern driven by both natural processes and human activities, rendering vast areas of land unsuitable for agriculture. According to the Food and Agriculture Organization, nearly 20% of the world’s irrigated land is already affected by salinity, highlighting the severity of the issue. Each year, approximately 10 Mha of productive agricultural land are lost due to increasing salt accumulation in soils (FAO, 2021). Several factors contribute to this problem, including climate change, excessive extraction of groundwater, and the use of low-quality irrigation water. Additionally, intensive irrigation in semi-arid and arid regions, combined with insufficient soil leaching, accelerates salt buildup (Kramer et al., 2025). Modern irrigation practices, while essential for crop production, are among the leading causes of rising salinity levels in both soils and underground water systems (Nicolas et al., 2023). In South Asia alone, annual economic loss due to salinization is estimated at US$1,500 million, severely affecting agriculture, livelihoods, food security, and sustainable development (Young, 1994).

Salinity stress affects plant growth by reducing water uptake and nutrient uptake and imposing toxicity to high sodium. Increased salt concentration adversely alters germination, seedling growth, leaf number, bulb diameter, bulb weight, and root growth (Sanwal et al., 2022). Salinity stress causes nutrient imbalance by increasing the intake of Na^+^ ions and concurrently declining the absorption of K^+^, Mg^2+^, and Ca^2+^ ions. This imbalance leads to augmentation of Na^+^ ions’ toxicity, which hampers the photosynthesis process by lowering the biosynthesis of photosynthetic pigments (Ashraf and Harris, 2013). Excessive salt stress stimulates oxygen poisoning by the production of reactive oxygen species (ROS) such as singlet oxygen, free oxygen radicals, and superoxide ions, which eventually leads to lipid oxidation of cellular membranes and damages lipid, proteins, nucleic acids, and finally normal cell functioning (Azeem et al., 2023). Moreover, salt stress impacts the photosynthesis phenomenon by restricting CO_2_ entry and assimilation rate through stomatal closure, disabling the electron transport chain (ETC) and regulation of stress-responsive genes (Alzahib et al., 2021). Plants use antioxidant systems such as catalase (CAT), superoxide dismutase (SOD), and ascorbate peroxidase (APX) to neutralize the harmful effect of oxidative stress. Salt stress tolerance in vegetable crops is linked to homeostasis of ions via exclusion and partitioning of Na^+^. Additionally, osmotic adjustment via synthesis and accumulation of proline and glycine-betaine plays a critical role in protection against osmotic stress by stabilizing the proteins’ tertiary structure (Kahlaoui et al., 2013).

Garlic (*Allium sativum* L.) is an important bulb and medicinal crop having high vitamin content, antioxidant activity, and sulfur-containing compounds such as allicin, which also imparts its characteristic flavor and makes it an integral part of many cuisines (Ammarellou et al., 2022; Pasupula et al., 2024). India is the second largest producer of garlic where it is cultivated across a wide range of temperatures and soil types in the regions of Madhya Pradesh, Rajasthan, Uttar Pradesh, Gujarat, Assam, Punjab, and Maharashtra (Sönmez et al., 2025**;** Sanwal et al., 2012). Despite the large cultivated area and high production of garlic in India, its productivity is five times less than that of China, Uzbekistan, and Egypt; poor soil fertility and poor quality of water and soil are among the important factors (Chhetri et al., 2024**).**

Garlic can tolerate soil salinity up to 3.9 dS m^-^¹ electrical conductivity and is classified as moderately salt-tolerant. The same study reported that irrigation with saline water with an EC of 8 dS m^-^¹ reduces the bulb yield by 50% (Nana et al., 2024). Previous reports by Talukder et al. (2021) have shown a 71.6% reduction in bulb yield at 12 dS m^-^¹ salinity level. Apart from bulb yield, salinity stress significantly reduced the bulb weight and bulb size at elevated level of salt concentration, which may be due to failure of garlic plants to synthesize photosynthetic pigments and ionic imbalance. They also observed genotypic differences for yield reduction at different levels of salinity, indicating the importance of genetic diversity in garlic improvement programs. Salinity stress primarily affects plant growth through accumulation of higher levels of Na^+^ and Cl^-^ ions in the root zone, inducing ion toxicity and osmotic stress in plant, causing physiological drought and finally disrupting normal physiological functions. It was observed that garlic genotypes exhibit variable responses to soil salinity, primarily due to a gradual decline in sprouting and shoot growth under increasing stress conditions. A marked reduction in yield occurs when electrical conductivity (ECe) reaches 4.0 dS m^−1^ or higher. However, garlic shows moderate tolerance, withstanding salinity levels between 5.60 and 7.80 dS m^−1^ depending on the cultivar, indicating the importance of genotype selection for saline environments (Mangal et al., 1990).

Considering the detrimental responses of salinity on bulb yield and quality, along with genotypic differences, provides a great opportunity for the identification suitable salinity-tolerant lines. Furthermore, identification of important traits associated with salinity tolerance and understanding the physiological and biochemical mechanism of tolerance will enable the development of garlic genotypes adaptable to saline stress conditions. Therefore, the present study was conducted to evaluate 36 genotypes of garlic under normal and saline conditions with objectives to assess genotypic differences in salinity tolerance and identify key morphological, physiological, and biochemical traits associated with tolerance that can be utilized in future garlic improvement programs.

## 2 Materials and methods

### 2.1 Experimental material and site

**Figure 1:**
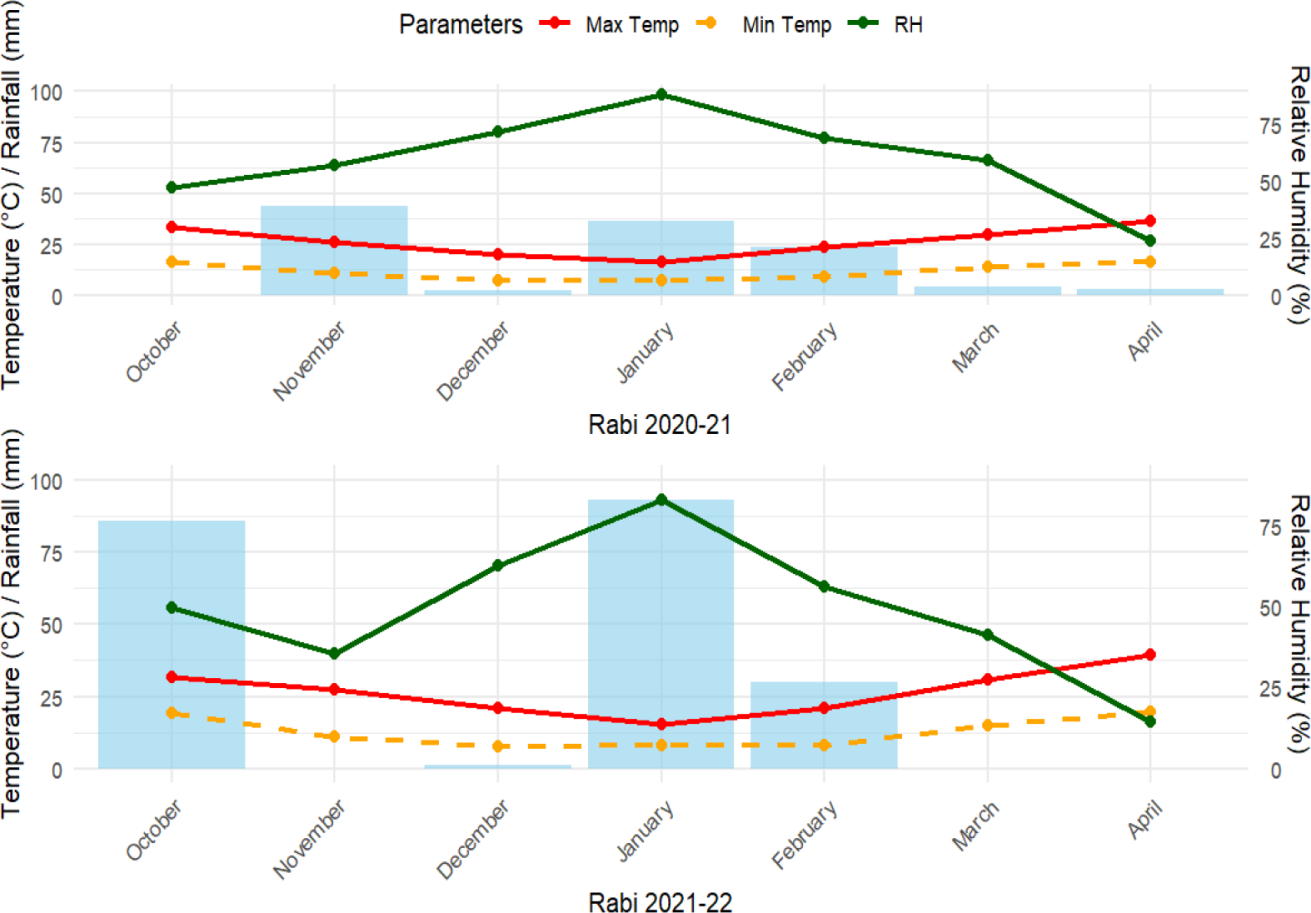
Precipitation distribution, temperatures, and relative humidity during the crop season of 2020–2021 and 2021–2022.

The experiment was conducted in micro-plots at the ICAR–Central Soil Salinity Research Institute (29 41' 24ʺ N, 76 59ʹ24ʺ E, 256 above sea level), Karnal (India), during the rabi seasons of 2020–2021 and 2021–2022. Micro-plots are small, covered concrete structures (typically 2 × 2 × 1 meters) designed for controlled experiments. They regulate water flow and salt movement, allowing precise study of soil conditions, irrigation effects, and salinity management. The climatic condition of the experimental area is humid sub-tropical, dry winter climate with an annual temperature and precipitation of 28.27 °C and 582 mm, respectively. The weather conditions during the two experimental years (Rabi seasons of 2020–2021 and 2021–2022) exhibited distinct variations, particularly concerning precipitation distribution and late-season temperatures. During the first Rabi season (October 2020 to April 2021), temperatures followed a standard winter decline, with maximum and minimum temperatures reaching their lowest in January (approximately 17 °C and 7 °C, respectively) before steadily increasing through April. Rainfall was relatively moderate and distributed, with primary peaks occurring in November (~43 mm) and January (~36 mm). Relative humidity showed a smooth bell-shaped curve, peaking at over 80% in January and declining to roughly 22% by April. In contrast, the second Rabi season (October 2021 to April 2022) experienced more erratic precipitation and higher late-season heat, with maximum April temperatures approaching 40 °C. Rainfall during this second year was highly skewed, marked by significant early moisture in October (>80 mm) and an exceptional mid-season deluge in January (nearly 100 mm), contrasted by prolonged dry spells in November, December, and March. Relative humidity mirrored these fluctuations, displaying a sharp dip in November, a sudden peak coinciding with the January rains (>80%), and a steep plummet to below 20% by the end of the season (**Figure 1**).

The 36 garlic accessions including advanced lines and released genotypes used in the study were procured from the National Horticulture Research and Development Foundation, Karnal, ICAR–Indian Agricultural Research Institute, New Delhi, India and the ICAR–Directorate of Onion and Garlic Research, Pune. The selection of garlic genotypes was based on regional adaptation, agronomic relevance, and potential for genetic diversity. The passport details of the tested genotypes can be seen in **Supplementary Table 1**.

### 2.2 Experimental design

Single-clove seed of garlic was planted at a spacing of 10 cm within rows in microplots under control and saline conditions (EC_iw_ ≈ 7 dS m^-^¹) in a randomized complete block design with three replications. Each row length was 2 m and accommodated 20 plants with a row-to-row spacing of 15 cm within each replication. To mimic farmers’ practices, salinity stress in garlic was imposed from 7 days after planting by applying saline irrigation (EC_iw_ ≈ 7 dS m^-^¹) throughout the crop growth period, covering all stages with 10 irrigations based on 100% crop evapotranspiration. Saline irrigation water (EC_iw_ ≈ 7 dS m^-^¹) was prepared by diluting naturally saline groundwater (EC_iw_ ≈ 18 dS m^-^¹), while the best available water (EC_iw_ ≈ 1.04 dS m^-^¹) was used for the control treatment (**Table 1**). Fertilizer doses were applied according to soil test-based recommendations. Fifty percent of nitrogen and 100% of potassium and phosphorus were applied as basal dose before planting. The remaining nitrogen was applied in two equal parts at 30 and 45 days after planting. Soil samples were collected from both control and saline plots prior to sowing and after harvest for soil analysis to see the salt accumulation in the experimental duration.

**Table 1:** Quality parameters of irrigation water.

Parameter	pH	Salinity (dS/m)	Ca + Mg (meq/L)	CO_3_ (meq/L)	HCO_3_ (meq/L)	Na (meq/L)	Chloride (meq/L)	SO_4_ (meq/L)	RSC	SAR
Best available water	7.93	0.8	5	Nil	6.2	3.47	0.8	1	1.2	2.2
Saline water	8.72	17.5–18.0	67	2	7	172	94	105.5	Nil	29.7

### 2.3 Morphological, physiological, and biochemical traits

The 36 genotypes were evaluated using various morphological, physiological, and biochemical traits. Morphological traits included yield per plant (g), bulb equatorial diameter (ED) (cm), bulb polar diameter (PD) (cm), number of cloves per bulb, plant height (PH) (cm), and total soluble solids (TSS, %). Bulb diameter was measured with an electronic Vernier calliper from five samples, and the individual average was taken in analysis. TSS were determined using a handheld refractometer as earlier done by Robert and Bradley (2010).

Physiological traits, *viz.*, membrane stability index (MSI), relative water content (RWC), photosynthetic rate (Pn), transpiration rate (E), and stomatal conductance (g_s_) were measured after 110 days of planting. Photosynthetic rate (μmol CO_2_ m^−2^ s^−1^), transpiration rate (E), and stomatal conductance (g_s_) were recorded from fully expanded third leaf apex using a portable system (LI-COR-6800, Inc., Lincoln, NE, USA). All the observations of photosynthesis were carried out on clear sunny morning hours prior to 11 a.m. Samples for Na^+^ and K^+^ concentration estimation in shoots, roots, and bulbs were taken at harvest time. Potassium (K^+^) content was estimated according to Ghosh et al. (1983) and sodium (Na^+^) content was determined following the method given by Jackson (1973). The extracts obtained from the respective plant parts were analyzed using a flame photometer (Systronics, India) and expressed as mg g^-^¹ dry weight.

Biochemical trait analysis of the plant samples was done after 110 days of planting for malondialdehyde (MDA), proline content (Pro), hydrogen peroxide (H_2_O_2_), and antioxidant enzyme activities such as CAT, APX, SOD, and peroxidase (POX). Samples were collected in the morning hours (9:00 a.m.) packed in labeled plastic bags and immediately placed in an icebox for enzyme assays. In the lab, samples were washed with distilled water, dried, and stored in aluminum foil at −80°C until they are crushed for further use. The lipid peroxidation was estimated by assessing MDA content using the method given by Zhang and Qu (2004). Proline content (mg/mL) was estimated using the ninhydrin colorimetric method (Yang et al., 2020). H_2_O_2_ content was determined using the protocol by Distéfano et al. (2008). The SOD, POX, CAT and APX activity was estimated as per the method described by Gao (2006); Hayat et al. (2018); Sanwal et al., 2022 and Nakano and Asada (1981), respectively.

### 2.4 Statistical analysis

All recorded parameters were systematically organized in a Microsoft Excel sheet according to thematic categories. To ensure the validity of statistical analysis, a test of normality for each parameter was conducted using the Shapiro–Wilk test, thereby confirming compliance with the assumptions required for analysis of variance (ANOVA). In cases where parameters violated normality assumptions, appropriate data transformation techniques were applied. Subsequently, a two-way ANOVA was employed to evaluate the effects of genotypes, salinity levels, and their interactions. Additionally, group comparisons between tolerant and sensitive cultivars were performed though *F*-test using the STAR software (IRRI, 2014). A paired *t*-test (Steel and Torrie, 1980) was applied to evaluate the significance of changes in the magnitude of morphological, physiological, and biochemical traits under salinity stress. This statistical method helped determine whether the observed differences before and after stress exposure were meaningful and not due to random variation in the studied samples.

Based on observations from the first-year trial, garlic accessions were classified into three categories according to bulb yield reduction under salinity stress relative to control conditions: tolerant (≤15%), moderately tolerant (15%–30%), and susceptible (≥30%). From this classification, six tolerant and five susceptible accessions were selected in the second-year experiment, for further investigation to understand the physio-biochemical mechanisms underlying salinity tolerance.

Bulb reduction percentage was estimated through the following equation:


$$\text{Percent Bulb reduction}=\frac{\left(\text{Bulb Yield under Control}-\text{Bulb Yield under Salinity}\right)}{\text{Bulb Yield under Control}}\times 100$$

A response equation to predict bulb yield under salinity stress was developed using a stepwise regression approach (backward elimination). Morphological, physiological, and biochemical traits showing significant association with bulb yield (*p* > 0.05) were identified and prioritized. These key traits will be useful for future studies on salinity stress tolerance in garlic. The analysis and model development were carried out using STAR software.

## 3 Results

### 3.1 Growth and bulb parameters affected by salt stress

ANOVA for morphological, bulb yield, TSS, and ionic traits of 36 garlic genotypes was analyzed separately under control and salinity stress (EC_iw_ 7 dS/m) conditions (**Table 2**). Under both treatments, year, genotypes, and genotypes × year interactions showed a highly significant effect (*p* ≤ 0.01) for most traits, indicating differential response of genotypes to the treatments as well as year. Significant genotypic effects for the traits (yield, bulb dimensions, PH, TSS, and Na^+^/K^+^ ratios) showed the presence of genetic variability among the studied genotypes for salt stress tolerance. Coefficient of variance (CV) accesses the relative variability of measured traits, where low CV indicates experimental reliability and homogeneity of data. Only bulb diameter showed a higher CV in both treatments, showing higher variability for the traits in the genotypes (**Table 3**). All the traits, except PH, showed a high CV under salt stress than control, showing a variable response of genotypes to salinity stress.

**Table 2:** ANOVA of morphological traits of 36 garlic cultivars under control and salinity stress.

Sources of variation	DF	Yield/plant (g)	No. of cloves/bulb	Polar diameter of bulb (cm)	Equatorial diameter of bulb (cm)	Plant height (cm)	TSS %	Bulb_ Na^+^/K^+^	Root_ Na^+^/K^+^	Shoot_ Na^+^/K^+^
Control condition										
Year	1	15.821^**^	33.037^**^	1.116^**^	0.765^**^	134.89^**^	2.153^**^	0.000^ns^	2.327^**^	0.004^**^
Genotypes	35	118.690^**^	95.195^**^	0.711^**^	0.917^**^	449.32^**^	13.84^**^	0.003^**^	0.476^**^	0.004^**^
Gen*Year	35	9.509^**^	5.057^**^	0.171^**^	0.178^**^	4.834^**^	2.257^**^	0.001^**^	0.205^**^	0.001^**^
Residuals	139	0.112	0.049	0.002	0.000	1.695	0.225	0.000	0.002	0.000
Salinity stress (EC_iw_ 7.0 dS/m)										
Year	1	9.259^**^	59.199^**^	0.026^**^	1.197^**^	61.12^**^	5.308^*^	0.000^**^	3.219^**^	0.000^ns^
Genotypes	35	94.485^**^	82.351^**^	0.468^**^	0.923^**^	522.28^**^	13.63^**^	0.006^**^	2.135^**^	0.008^**^
Gen*Year	35	3.225^**^	2.534^**^	0.064^**^	0.163^**^	57.87^**^	2.446^**^	0.001^**^	0.416^**^	0.003^**^
Residuals	139	0.444	0.458	0.001	0.001	0.96	0.005	0.000	0.018	0.000

ns, non-significant; * significant at *p* ≥ 0.05; ** significant at *p* ≥ 0.01.

**Table 3:** Mean comparisons of morphological traits of garlic cultivars under control and salinity stress.

Cultivar name	Yield/plant (g)		No. of cloves/bulb		Polar diameter of bulb (cm)	
	Cont.	Sal.	Cont.	Sal.	Cont.	Sal.
Selection-459	18.90 ± 1.04	8.65 ± 0.57	21.15 ± 0.60	15.98 ± 1.43	3.69 ± 0.59	3.09 ± 0.12
Agrifound White (G-41)	17.42 ± 1.47	11.59 ± 1.38	24.60 ± 1.04	21.30 ± 0.76	3.95 ± 0.64	3.35 ± 0.33
Yamuna Safed-4 (G-323)	22.87 ± 1.02	18.15 ± 0.75	26.75 ± 2.09	23.80 ± 1.48	4.04 ± 0.24	3.49 ± 0.08
Yamuna Safed-2 (G50)	19.46 ± 0.85	15.33 ± 0.39	19.10 ± 1.72	17.30 ± 1.43	3.81 ± 0.32	3.53 ± 0.05
Yamuna Safed-5 (G-189)	21.46 ± 0.64	14.76 ± 0.87	29.60 ± 2.31	24.90 ± 1.82	3.71 ± 0.09	3.55 ± 0.03
Yamuna Safed-8 (G-384)	23.35 ± 1.72	20.28 ± 0.89	25.35 ± 0.65	19.65 ± 1.34	4.55 ± 0.39	3.73 ± 0.06
Bhima Purple	20.89 ± 1.40	16.73 ± 1.03	22.05 ± 2.27	19.10 ± 2.11	3.97 ± 0.23	3.26 ± 0.12
Bhima Omkar	10.07 ± 1.52	6.33 ± 1.14	20.55 ± 0.61	16.95 ± 2.52	3.08 ± 0.30	2.59 ± 0.48
Yamuna Safed-9 (G-386)	18.99 ± 1.72	13.40 ± 1.12	11.25 ± 0.41	10.80 ± 0.41	3.90 ± 0.20	2.99 ± 0.19
Yamuna Purple10(G-404)	24.68 ± 1.30	21.90 ± 1.03	22.75 ± 0.76	20.80 ± 0.42	4.08 ± 0.18	3.70 ± 0.12
Yamuna Safed (G-1)	26.17 ± 1.35	22.75 ± 1.07	26.15 ± 1.76	21.95 ± 1.60	4.35 ± 0.37	3.48 ± 0.09
Yamuna Safed-3 (G-282)	24.86 ± 1.95	22.22 ± 0.83	24.50 ± 2.77	24.00 ± 2.52	4.14 ± 0.07	3.56 ± 0.10
G-324	23.00 ± 0.43	19.95 ± 0.69	23.05 ± 0.62	21.75 ± 0.77	3.80 ± 0.06	3.58 ± 0.05
G-304	22.70 ± 0.27	20.15 ± 0.54	22.05 ± 0.47	20.80 ± 0.34	3.96 ± 0.05	3.39 ± 0.08
G-363	24.05 ± 0.45	19.10 ± 0.46	20.65 ± 0.42	19.40 ± 0.53	3.83 ± 0.05	3.53 ± 0.04
G-378	18.55 ± 0.57	15.15 ± 0.94	13.31 ± 0.52	12.00 ± 0.63	3.56 ± 0.04	3.27 ± 0.04
PGS-200	18.95 ± 0.63	14.65 ± 0.86	26.80 ± 0.62	23.55 ± 0.79	3.59 ± 0.06	3.28 ± 0.07
PGS-201	15.75 ± 0.55	12.22 ± 0.80	25.90 ± 0.55	24.83 ± 0.45	3.38 ± 0.06	3.02 ± 0.04
PGS-202	16.15 ± 0.60	11.87 ± 0.68	21.65 ± 0.51	20.55 ± 0.68	3.51 ± 0.04	3.05 ± 0.05
PGS-203	14.31 ± 0.39	11.55 ± 0.63	19.05 ± 0.42	17.15 ± 0.45	3.46 ± 0.05	2.99 ± 0.03
PGS-204	16.10 ± 0.38	13.20 ± 1.13	21.60 ± 0.53	20.20 ± 0.34	3.54 ± 0.05	3.16 ± 0.02
PGS-205	16.75 ± 0.46	12.10 ± 0.90	26.00 ± 0.80	25.10 ± 0.92	3.56 ± 0.06	3.22 ± 0.03
PGS-206	17.95 ± 0.27	14.70 ± 0.75	27.10 ± 0.55	25.65 ± 0.40	3.56 ± 0.05	3.32 ± 0.02
PGS-207	17.75 ± 0.37	14.33 ± 0.91	23.55 ± 0.48	22.45 ± 0.72	3.32 ± 0.05	3.02 ± 0.03
PGS-208	13.55 ± 0.73	8.43 ± 1.34	17.95 ± 0.45	16.30 ± 1.35	3.37 ± 0.05	2.61 ± 0.02
PGS-209	12.20 ± 0.48	10.20 ± 0.57	20.35 ± 0.55	18.63 ± 0.42	3.24 ± 0.05	2.97 ± 0.03
PGS-210	19.95 ± 0.60	16.85 ± 0.87	24.35 ± 0.52	20.45 ± 0.74	3.88 ± 0.05	3.35 ± 0.06
PGS-211	15.00 ± 0.40	12.03 ± 0.73	20.60 ± 0.53	19.60 ± 0.78	3.76 ± 0.05	3.47 ± 0.05
PGS-212	15.35 ± 0.57	11.88 ± 0.95	16.30 ± 0.44	15.05 ± 0.71	3.51 ± 0.06	3.08 ± 0.03
PGS-215	17.30 ± 0.58	13.95 ± 0.79	26.88 ± 0.62	25.80 ± 0.43	3.62 ± 0.04	3.29 ± 0.02
PGS-216	21.15 ± 0.39	18.23 ± 0.90	27.90 ± 0.44	25.58 ± 0.58	4.14 ± 0.06	3.35 ± 0.02
PGS-217	18.35 ± 0.29	14.65 ± 1.11	20.65 ± 0.50	18.55 ± 0.46	3.48 ± 0.05	3.16 ± 0.04
GG-2	12.05 ± 0.51	7.55 ± 0.30	22.10 ± 0.49	20.90 ± 0.27	3.00 ± 0.06	2.94 ± 0.03
GG-4	14.80 ± 0.29	10.92 ± 0.43	27.35 ± 0.52	24.70 ± 0.69	3.25 ± 0.04	2.83 ± 0.04
Godawari	16.10 ± 0.18	13.01 ± 0.45	23.30 ± 0.49	21.85 ± 0.45	3.42 ± 0.05	3.05 ± 0.04
Phule Baswant	17.00 ± 0.37	12.17 ± 0.66	21.55 ± 0.62	20.80 ± 1.06	3.88 ± 0.05	3.01 ± 0.04
LSD (5%)	2.48	3.51	2.48	2.48	2.48	2.48
CV	5.53	9.78	4.42	4.88	27.10	30.98

Cultivar name	Equatorial diameter of bulb (cm)		Plant height (cm)		TSS%	
	Cont.	Sal.	Cont.	Sal.	Cont.	Sal.
Selection-459	2.83 ± 0.43	2.69 ± 0.37	66.17 ± 1.42	49.13 ± 1.27	41.70 ± 1.44	39.85 ± 1.48
Agrifound White (G-41)	3.29 ± 0.71	2.86 ± 0.62	76.97 ± 1.23	51.96 ± 1.12	40.12 ± 1.53	37.13 ± 0.96
Yamuna Safed-4 (G-323)	3.82 ± 0.06	3.70 ± 0.13	97.10 ± 1.92	79.32 ± 1.05	43.81 ± 0.88	41.83 ± 1.47
Yamuna Safed-2 (G50)	3.30 ± 0.21	3.29 ± 0.45	86.47 ± 1.53	71.09 ± 1.34	44.56 ± 0.66	41.32 ± 0.69
Yamuna Safed-5 (G-189)	3.71 ± 0.20	3.48 ± 0.40	88.10 ± 1.70	68.85 ± 1.23	42.80 ± 1.22	39.68 ± 1.09
Yamuna Safed-8 (G-384)	3.97 ± 0.32	3.83 ± 0.31	88.82 ± 1.30	69.68 ± 1.31	39.77 ± 1.74	37.25 ± 2.10
Bhima Purple	3.60 ± 3.60	3.09 ± 0.08	78.61 ± 1.71	48.85 ± 1.56	43.76 ± 1.99	40.27 ± 0.18
Bhima Omkar	2.32 ± 0.19	2.26 ± 0.35	58.51 ± 1.72	42.71 ± 1.15	38.46 ± 0.67	35.45 ± 0.72
Yamuna Safed-9 (G-386)	3.35 ± 0.55	2.75 ± 0.25	71.65 ± 1.31	56.47 ± 1.54	43.73 ± 0.59	40.70 ± 0.34
Yamuna Purple10(G-404)	3.68 ± 0.09	3.55 ± 0.21	88.45 ± 1.51	58.95 ± 1.97	44.00 ± 0.78	42.00 ± 0.24
Yamuna Safed (G-1)	4.03 ± 0.37	3.51 ± 0.12	92.37 ± 2.03	78.67 ± 1.40	45.58 ± 1.00	42.33 ± 1.68
Yamuna Safed-3 (G-282)	3.71 ± 0.03	3.68 ± 0.38	85.63 ± 2.38	77.57 ± 1.19	43.00 ± 0.55	41.00 ± 1.32
G-324	3.78 ± 0.02	3.44 ± 0.05	87.95 ± 1.31	78.80 ± 1.10	42.72 ± 0.22	41.53 ± 0.42
G-304	3.63 ± 0.02	3.13 ± 0.06	85.76 ± 1.61	62.33 ± 2.41	42.05 ± 0.33	40.90 ± 0.13
G-363	3.50 ± 0.02	3.08 ± 0.05	89.02 ± 1.75	69.34 ± 2.65	43.20 ± 0.53	40.17 ± 0.31
G-378	3.38 ± 0.07	3.31 ± 0.06	89.59 ± 1.38	71.89 ± 1.55	40.59 ± 0.77	38.69 ± 0.25
PGS-200	3.42 ± 0.02	3.13 ± 0.04	84.20 ± 1.49	71.85 ± 2.16	42.84 ± 0.46	40.15 ± 0.10
PGS-201	3.27 ± 0.02	2.78 ± 0.05	86.72 ± 1.59	63.46 ± 1.54	41.15 ± 0.37	40.09 ± 0.07
PGS-202	3.22 ± 0.05	2.91 ± 0.04	90.53 ± 3.28	69.31 ± 1.44	42.27 ± 0.60	39.81 ± 0.17
PGS-203	3.46 ± 0.06	3.00 ± 0.05	84.08 ± 2.00	59.32 ± 2.89	40.54 ± 0.51	39.66 ± 0.11
PGS-204	3.51 ± 0.03	3.15 ± 0.05	84.44 ± 1.54	69.32 ± 1.09	42.83 ± 0.54	40.73 ± 0.15
PGS-205	3.28 ± 0.02	2.92 ± 0.05	85.50 ± 1.35	67.88 ± 1.96	43.07 ± 0.53	41.06 ± 0.26
PGS-206	3.47 ± 0.05	3.21 ± 0.05	86.31 ± 1.19	69.79 ± 2.97	43.12 ± 0.68	41.64 ± 0.22
PGS-207	3.68 ± 0.03	3.23 ± 0.06	84.80 ± 1.38	72.41 ± 2.35	40.96 ± 0.43	39.44 ± 0.14
PGS-208	2.87 ± 0.02	2.41 ± 0.04	74.53 ± 1.53	58.18 ± 1.56	42.30 ± 0.54	40.32 ± 0.21
PGS-209	2.87 ± 0.02	2.59 ± 0.04	67.70 ± 1.43	55.09 ± 1.13	42.98 ± 0.19	42.14 ± 0.44
PGS-210	3.73 ± 0.02	3.21 ± 0.05	82.62 ± 2.20	66.08 ± 2.95	42.03 ± 0.26	41.00 ± 0.20
PGS-211	2.93 ± 0.04	2.71 ± 0.04	74.62 ± 1.66	59.71 ± 1.11	43.39 ± 0.56	41.51 ± 0.07
PGS-212	2.96 ± 0.03	2.77 ± 0.05	70.16 ± 1.47	54.56 ± 1.46	42.18 ± 0.41	40.61 ± 0.07
PGS-215	3.27 ± 0.08	3.22 ± 0.05	84.67 ± 1.25	63.90 ± 2.76	44.74 ± 0.39	41.01 ± 0.38
PGS-216	3.97 ± 0.08	3.77 ± 0.06	80.65 ± 1.39	63.94 ± 1.95	43.45 ± 0.38	42.17 ± 0.10
PGS-217	3.55 ± 0.05	2.86 ± 0.06	75.61 ± 1.48	58.39 ± 1.52	42.61 ± 0.67	39.12 ± 0.57
GG-2	2.72 ± 0.03	2.38 ± 0.05	67.98 ± 1.58	51.14 ± 2.17	39.68 ± 0.80	38.21 ± 0.42
GG-4	2.97 ± 0.02	2.80 ± 0.04	74.18 ± 2.49	52.93 ± 2.09	42.92 ± 0.41	39.86 ± 0.22
Godawari	2.97 ± 0.02	2.86 ± 0.04	82.25 ± 2.06	58.58 ± 1.11	43.25 ± 0.22	41.35 ± 0.19
Phule Baswant	3.31 ± 0.02	2.93 ± 0.05	69.98 ± 2.29	57.14 ± 1.08	42.10 ± 0.56	40.37 ± 0.50
LSD (5%)	2.48	2.48	3.51	2.48	2.48	2.48
CV	29.69	32.60	1.74	1.58	2.35	2.48

Salinity stress significantly influenced the morphological parameters across the 36 garlic genotypes. As shown in **Table 3**, yield/plant (g), no. of clove/bulb, PD (cm), ED (cm), PH (cm), and TSS (%) all decreased remarkably. The minimum yield reduction was noticed in Yamuna Safed-8 (9.26%) followed by Yamuna Safed-3 (10.62%), G-304 (11.23%), and Yamuna Purple-10, while maximum reduction was observed in Selection-459 (54.23%). The genotype Selection-459 showed maximum reduction (24.44%) for no. of cloves/bulb while the minimum was observed in Yamuna Safed-3 (G-282). For PD, ED, PH, and TSS, the highest reduction was observed in Yamuna Safed-9 (23.33%), PGS-217 (19.44%), Bhima Purple (37.86%), and PGS-215 (8.34%) while the lowest reduction was seen in GG-2 (2%), Yamuna Safed-2 (0.30%), Yamuna Safed-3 (9.41%), and PGS-209 (1.95%), respectively. Based on the % bulb yield reduction, the 36 genotypes were classified into three categories: (1) salt-tolerant genotypes having ≤15% yield reduction, (2) moderately salt-tolerant genotypes with 15%–30% yield reduction, and (3) salt-sensitive genotypes having >30% yield reduction (**Table 4**).

**Table 4:** Grouping of garlic cultivars based on % bulb yield reduction under salinity stress.

Salt-tolerant genotypes	Bulb yield reduction (<15%)	Moderately tolerant genotypes	Bulb yield reduction (15%–30%)	Salt-sensitive genotypes	Bulb yield reduction (>30%)
Yamuna Safed-8 (G-384)	9.26	PGS-210	15.54	Yamuna Safed-5 (G-89)	31.22
Yamuna Safed-3 (G-282)	10.62	PGS-209	16.39	Agrifound White (G-41)	33.47
G-304	11.23	PGS-204	18.01	Bhima Omkar	37.14
Yamuna Purple10 (G-404)	11.26	PGS-206	18.11	GG-2	37.34
Yamuna Safed (G-1)	13.07	G-378	18.33	PGS-208	37.79
G-324	13.26	Yamuna Safed-4 (G-323)	19.11	Selection-459	54.23
PGS-216	13.81	Godawari	19.19		
		PGS-207	19.27		
		PGS-203	19.29		
		PGS-215	19.36		
		PGS-211	19.80		
		Bhima Purple	19.91		
		PGS-217	20.16		
		G-363	20.58		
		Yamuna Safed-2 (G50)	21.22		
		PGS-201	22.41		
		PGS-212	22.61		
		PGS-200	22.69		
		GG-4	26.22		
		PGS-202	26.50		
		PGS-205	27.76		
		Phule Baswant	28.41		
		Yamuna Safed-9 (G-386)	29.44		

### 3.2 Responses of morphological, physiological, and biochemical traits to salinity stress

The test of significance for percent deviation in various traits of 36 garlic genotypes is given in **Table 5** (*p* < 0.001). Salinity significantly reduced morphological traits, with the highest reduction recorded for PH (22.04%) and bulb yield per plant (20.06%), followed by polar (12.51%), no. of cloves (9.29%), equatorial bulb diameter (8.94%), and TSS (5.10%). Physiological traits were also affected by salinity stress, as significant reductions were observed in stomatal conductance (15.83%), photosynthetic rate (15.69%), MSI (15.22%), transpiration rate (14.14%), and RWC (6.85%), indicating impaired membrane integrity and gas exchange. In contrast, biochemical stress indicators like Na^+^/K^+^ ratios in roots, shoots, and bulbs; proline; hydrogen peroxide; MDA; and antioxidant enzyme activities (CAT, APX, SOD, and POX) increased significantly.

**Table 5:** Test of significance for % change in morphological, physiological, and biochemical traits under salinity stress.

S. no.	Traits	Control	Salinity	Magnitude of change under salinity stress (%)		*t*-value	*Pr*(>|*t*|)
A	Morphological traits						
	Yield/plant (g)	18.09	14.46		20.06	19.29	0.000
	Equatorial diameter of bulb (cm)	3.37	3.07		8.94	10.21	0.000
	Polar diameter of bulb (cm)	3.69	3.23		12.51	12.77	0.000
	No. of cloves/bulb	22.61	20.50		9.29	9.36	0.000
	Plant height (cm)	81.18	63.29		22.04	21.68	0.000
	TSS %	42.45	40.29		5.10	15.51	0.000
B	Physiological traits						
	Relative water content (RWC)*	80.41	74.89		6.85	7.38	0.000
	Membrane stability index (MSI)*	83.39	70.69		15.22	11.51	0.000
	Photosynthesis rate (Pn)*	18.73	15.79		15.69	8.42	0.000
	Transpiration rate (E)*	5.24	4.50		14.14	9.36	0.000
	Stomatal conductance (g_s_)*	0.42	0.35		15.83	15.19	0.000
C	Biochemical traits						
	Root Na^+^/K^+^	1.03	2.50	141.77		−14.59	0.000
	Shoot Na^+^/K^+^	0.11	0.23	119.59		−20.78	0.000
	Bulb Na^+^/K^+^	0.11	0.17	55.17		−11.57	0.000
	Proline content (Pro)*	0.49	1.04	110.38		−15.34	0.000
	Hydrogen peroxide (H_2_O_2_)*	1.44	1.54	6.55		−8.22	0.000
	Malondialdehyde (MDA)*	15.50	18.61	20.05		−9.74	0.000
	Catalase (CAT)*	0.20	0.44	116.14		−7.74	0.000
	Ascorbate peroxidase (APX)*	81.43	124.24	52.56		−11.34	0.000
	Super oxide dismutase (SOD)*	143.70	221.78	54.34		−18.52	0.000
	Peroxidase (POX)*	25.45	59.15	132.47		−22.42	0.000
	Proline content (Pro)*	0.49	1.04	110.38		−32.98	0.000

*Traits observations recorded only in tolerant and sensitive genotypes identified in the first-year trial observations.

### 3.3 Salinity stress effect on garlic-tolerant and -sensitive genotypes

The effect of salinity stress on physiological and biochemical traits of garlic genotypes distinguished tolerant and sensitive genotypes (**Table 6**). The salt-tolerant group showed higher RWC and MSI as compared to the sensitive genotypes, indicating better water retention and cellular integrity of the tolerant group under salt stress. Photosynthetic performance was also better in the tolerant group as reflected by significantly higher Pn (17.3 µmol CO_2_ m^−2^ s^−1^) and g_s_ (0.38 mol H_2_O m^−2^ s^−1^) as compared to the sensitive group as shown in **Table 6**. Likewise, proline accumulation was significantly higher in the tolerant group (1.22 µg g^−1^ FW) than in the sensitive group (0.82 µg g^−1^ FW). In contrast, oxidative stress markers H_2_O_2_ and MDA were found to be significantly higher in the sensitive group, while the activities of antioxidant enzymes CAT, SOD, and POX were significantly higher in the tolerant group of genotypes.

**Table 6:** Effects of salinity stress on physiological and biochemical traits of selected salt-tolerant and sensitive genotypes.

Trait	Unit	Group mean		% Change	Mean square	Significance
		Tolerant genotypes	Sensitive genotypes		Tolerant vs. sensitive	*Pr*(>*F*)
RWC	%	77.34*^a^*	71.96*^b^*	7.48	237.14	0.000
MSI	%	75.25*^a^*	65.23*^b^*	15.36	821.51	0.000
Pn	µmol CO_2_ m^−2^ s^−1^	17.30*^a^*	13.98*^b^*	23.75	89.82	0.000
E	mmol H_2_O m^−2^ s^−1^	4.81*^a^*	4.12*^a^*	16.75	3.85	0.160
g_s_	mol H_2_O m^−2^ s^−1^	0.38*^a^*	0.32*^b^*	18.75	0.022	0.000
Proline	µg g^−1^ FW	1.22*^a^*	0.82*^b^*	48.78	1.34	0.000
H_2_O_2_	µmol g^−1^ FW	1.51*^b^*	1.57*^a^*	−3.82	0.031	0.000
MDA	nmol g^−1^ FW	17.05*^b^*	20.47*^a^*	−16.71	95.88	0.000
CAT	units g^−1^ FW	0.53*^a^*	0.33*^b^*	60.61	0.31	0.000
APX	units g^−1^ FW	136.53*^b^*	109.49*^a^*	24.70	5,985.18	0.000
SOD	units g^−1^ FW	232.62*^a^*	208.78*^b^*	11.42	4,648.80	0.000
POX	units g^−1^ FW	63.55*^a^*	53.88*^b^*	17.95	764.58	0.000

RWC, relative water content; MSI, membrane stability index; Pn, photosynthesis rate; E, transpiration rate; g_s_, stomatal conductance; Pro, proline content; H_2_O_2_, hydrogen peroxide; MDA, malondialdehyde; CAT, catalase; APX, ascorbate peroxidase; SOD, superoxide dismutase; POX, peroxidase.

#### 3.3.1 Physiological responses

**Figure 2:**
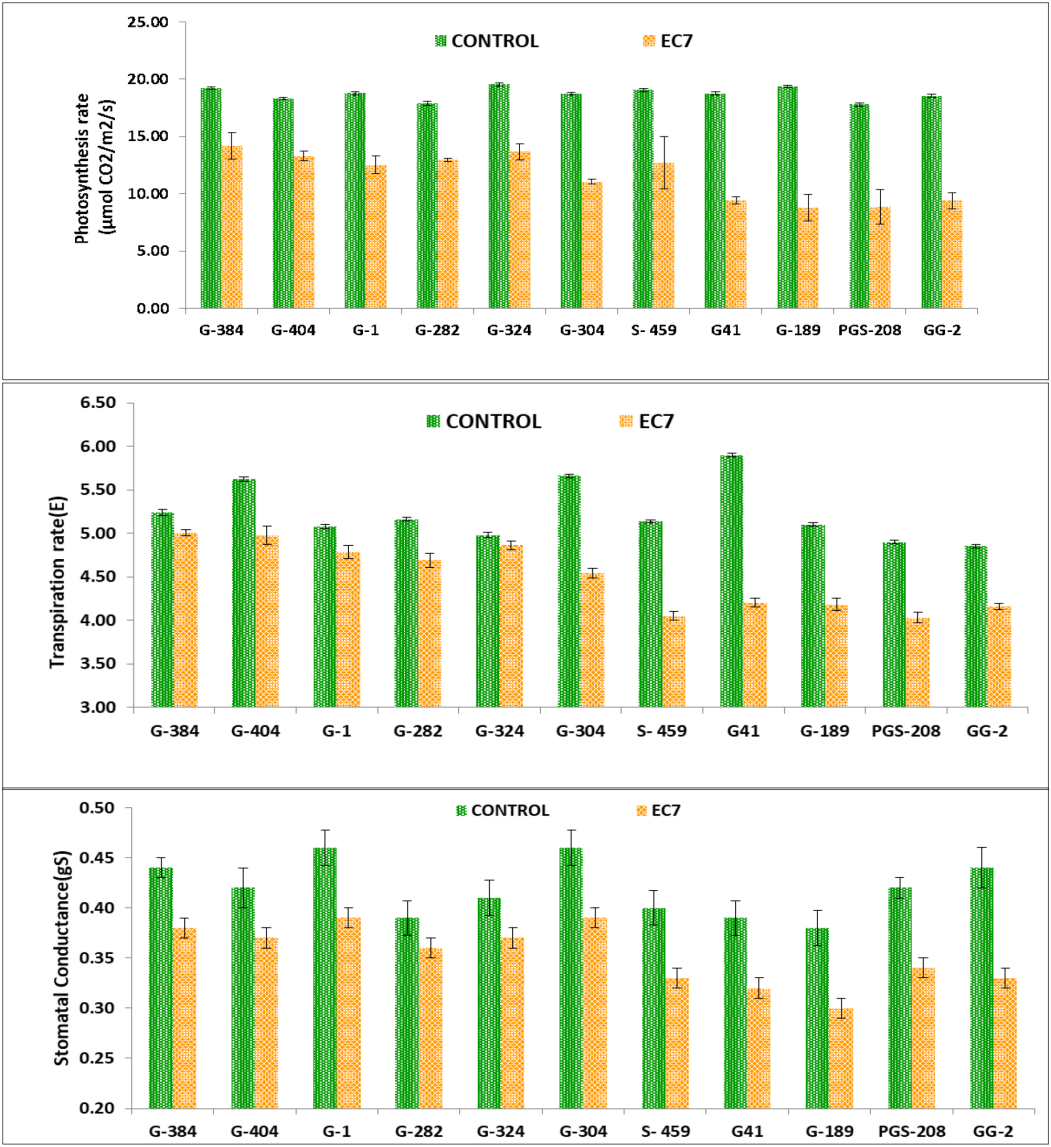
Gas exchange traits (photosynthetic rate), transpiration rate, and stomatal conductance of garlic cultivars under control and salinity stress (EC_iw_ 7 dS/m).

The response of six salt-tolerant (G-304, G-384, G-1, G-282, G-324, and G-404) and five salt-sensitive (G-41, S-459, PGS-208, GG-2, and G-89) garlic genotypes for photosynthetic rate (Pn), transpiration rate (E), and stomatal conductance (g_s_) under control and salinity stress (EC7) conditions is given in **Figure 2**. Although photosynthetic rate declined in all the genotypes under salinity stress, tolerant lines such as G-384, G-404, G-324, and G-304 maintained relatively higher Pn values under EC7, indicating their better photosynthetic stability. Similar results were observed for transpiration rate (E) with minimal relative change in genotypes G-324 and G-384, suggesting efficient water regulation under salt stress conditions. Stomatal conductance (g_s_) also decreased under salinity stress in all genotypes, but the tolerant group exhibited smaller reductions, indicating sustained stomatal functioning in tolerant genotypes under saline conditions.

#### 3.3.2 Biochemical responses

**Figure 3:**
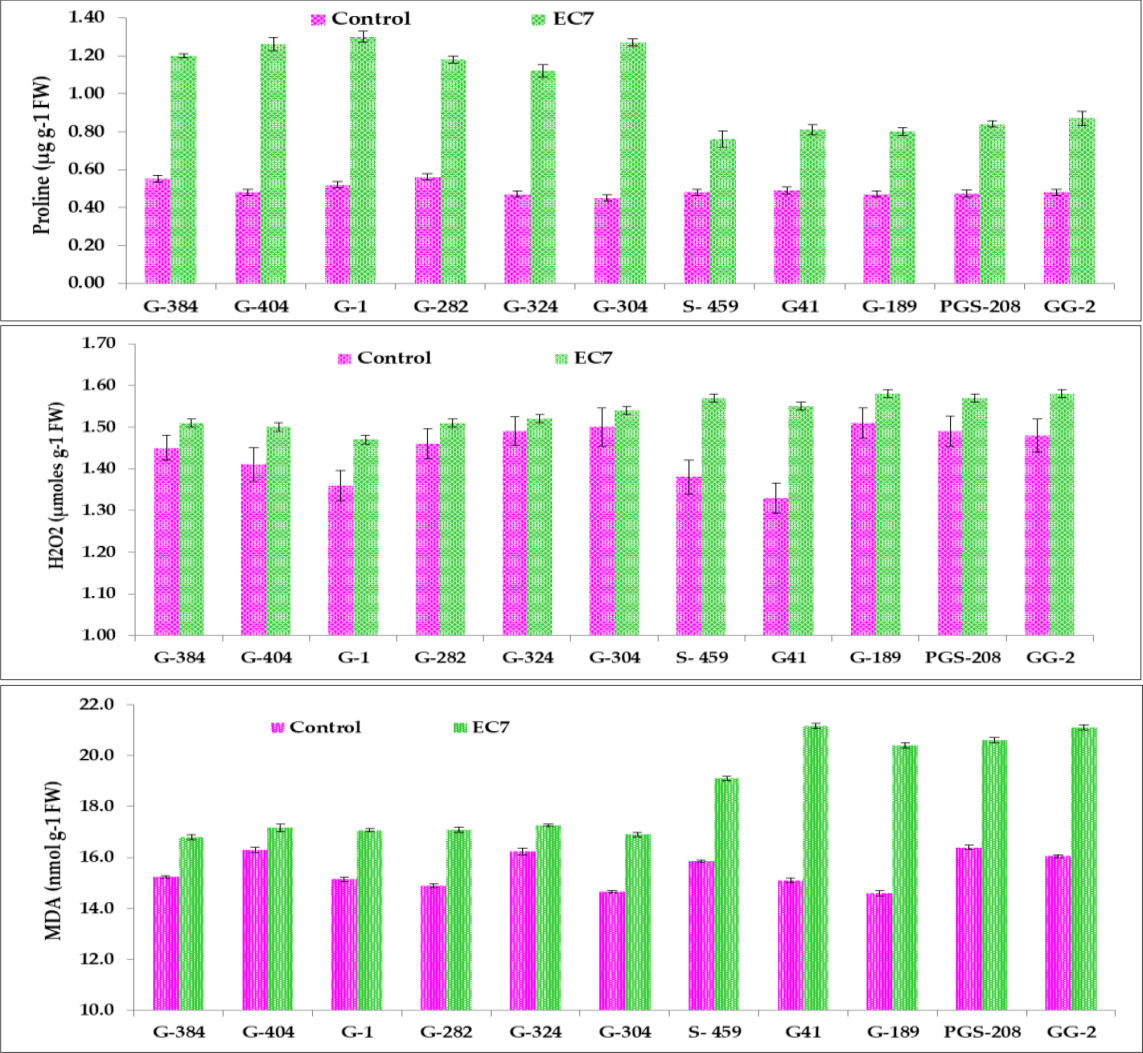
Biochemical parameters proline, H_2_O_2_, and MDA of selected garlic cultivars under control and salinity stress.

Proline accumulation increased markedly under salinity in all the genotypes. However, the tolerant genotypes showed the highest relative increase as compared to sensitive genotypes, indicating effective osmotic adjustment under stress. The maximum increase in proline content was observed in G1 and a minimum increase was observed in S-459. The increment in H_2_O_2_ content was observed in all 11 garlic genotypes; however, the accumulation of H_2_O_2_ is relatively high in sensitive genotypes in comparison to tolerant ones (up to 16.5%). For example, the maximum increase was observed in G-41, a salt-sensitive genotype, while a minimum increment was seen in G-324, a tolerant one. Likewise, MDA levels increased substantially in all the garlic genotypes. Sensitive genotypes such as G41, G189, S-459, PGS-208, and GG-2 recorded the highest increase in MDA accumulation, i.e., 25%–40% compared with genotypes G-404 and G-324 (**Figure 3**).

**Figure 4:**
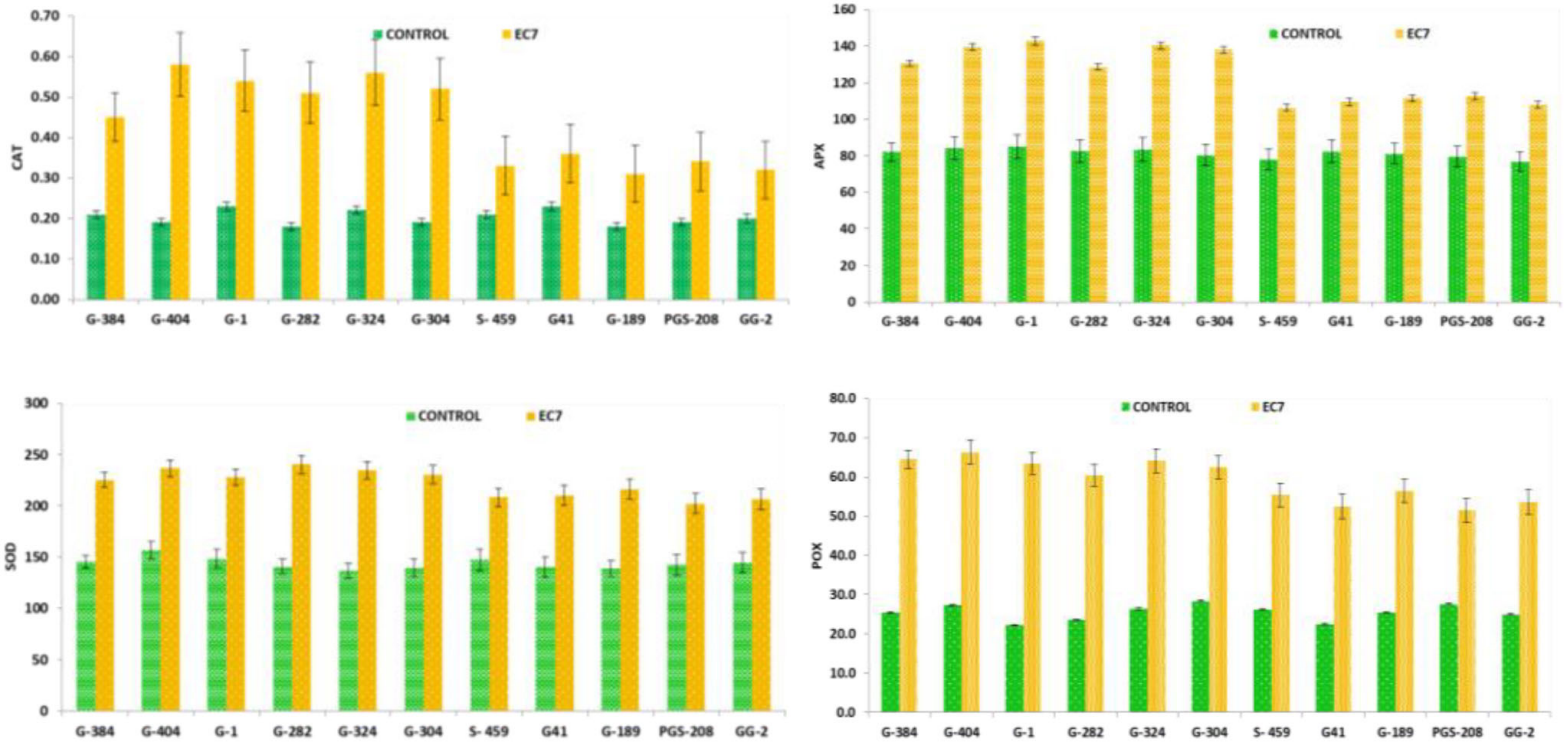
Antioxidative enzyme activity (CAT, APX, SOD, and POX) of selected garlic cultivars under control and salinity stress.

Salinity stress significantly enhanced the activities of CAT, APX, SOD, and POX, with a higher increase observed in salt-tolerant genotypes (**Figure 4**). CAT was observed to be higher in tolerant genotypes G-404, G-282, G-304, and G-324 as compared to sensitive genotypes. Higher levels of APX activity in tolerant genotypes indicate efficient detoxification of hydrogen peroxide. SOD activity showed pronounced enhancement in tolerant genotypes G-404 and G-282 under salt stress, reflecting improved superoxide radical scavenging. Similarly, POX activity increased markedly in tolerant genotypes (G-404 and G-324), having higher activity than sensitive genotypes.

### 3.4 Identification of traits associated with bulb yield in control and salinity stress

Data recorded for morphological and ionic traits were used to determine the relationship with bulb yield under control and salinity stress. The trait–bulb yield relationship was examined separately in control and salt stress in this study. Stepwise regression analysis was employed to identify the traits that made significant contributions to bulb yield variation in the 36 genotypes. Under control conditions, ED alone explained 72.28% of variation in yield. Inclusion of PH and PD improved the model, with the combined effect of ED, PD, and PH accounting for 81.69% of variability in bulb yield. Inclusion of ionic traits (Na^+^/K^+^ ratios of root, shoot and bulb) and TSS in the model resulted only in marginal improvements, explaining 83.68% of bulb yield variation, suggesting very little variation in physiological trait performance of genotypes under non-stress optimal conditions (**Table 7**). Under salinity stress, the most important contributor to yield variation in genotypes was ED explaining 64.22% of yield variation, although its relative contribution was reduced as compared to control conditions (**Table 8**). Under salinity stress, TSS and Na^+^/K^+^ ratios of root, shoot, and bulb entered the regression model and contributed substantially under salinity stress. The final model having ED, PD, TSS, and Na^+^/K^+^ in roots (R_NaK) explained 75.69% of yield variation, indicating that yield determination in salinity stress depends on a combination of bulb parameters and ionic homeostasis in roots. It is more practical to restrict selection criteria to ED, PD, TSS, and R_NaK, as including additional traits can significantly raise the overall cost of a breeding program without delivering a comparable improvement in explanatory effectiveness. Focusing on these four key traits provides a balanced and efficient approach, ensuring meaningful selection outcomes while maintaining resource efficiency. From a practical perspective, this streamlined set of traits is sufficient and offers a more consistent and coherent framework for effective selection decisions.

**Table 7:** Traits priority for garlic bulb yield under control conditions.

Dependent variable	Step and variables	*C*(*p*)	*R* ^2^	Adjusted *R*^2^
Bulb yield	ED	139.506	72.28	72.15
	PD + PH	65.917	78.24	78.04
	**ED + PD + PH**	**24.172**	**81.69**	**81.43**
	ED + PD + PH + B_NaK	8.685	83.07	82.75
	ED+ PD + PH + TSS + B_NaK	5.830	83.45	83.06
	ED + PD + PH + TSS + S_NaK + B_NaK	5.652	83.63	83.16
	ED + PD + PH + TSS + R_NaK + S_NaK + B_NaK	7.003	83.68	83.13
	ED + PD + NCB +PH +TSS + R_NaK + S_NaK+ B_NaK	9.000	83.68	83.05

Bold data show the best-fitted model.

**Table 8:** Traits priority for garlic bulb yield under salinity stress.

Dependent variable	Step and variables	*C*(*p*)	*R* ^2^	Adjusted *R*^2^
Bulb yield	ED	130.187	64.22	64.05
	ED + PD	59.789	71.79	71.52
	ED + PD + TSS	37.855	74.29	73.93
	**ED + PD + TSS + R_NaK**	**26.500**	**75.69**	**75.23**
	ED + PD+ TSS + R_NaK + S_NaK	16.744	76.92	76.37
	ED + PD + TSS + R_NaK + S_NaK + B_NaK	13.129	77.50	76.86
	ED + PD + PH + TSS + R_NaK + S_NaK + B_NaK	9.597	78.08	77.34
	ED + PD +NCB +PH +TSS + R_NaK + S_NaK+ B_NaK	9.000	78.35	77.52

*Bold data show the best-fitted model. ED, equatorial diameter of bulb; PD, polar diameter of bulb; NCB, no. of cloves/bulb; PH, plant height; TSS, total soluble solids; R_NaK, Na^+^/K^+^ in roots; S_NaK, Na^+^/K^+^ in shoots; B_NaK, Na^+^/K^+^ in bulbs.

## 4 Discussion

In current research, salt stress causes a significant reduction in PH, photosynthetic rate, transpiration rate, stomatal conductance, bulb yield, and other related parameters of garlic. A similar pattern was observed in the studies conducted by Zhou et al. (2021) and Ahmad et al. (2021). Salt stress impacts the morpho-physiological parameters of garlic by causing oxidative stress, ionic toxicity, and nutrient imbalance, which ultimately reduces plant growth and bulb yield (Bandehagh and Taylor, 2020). Furthermore, excessive accumulation of Na^+^, Cl^-^, and K^+^ in the plant root, shoot, and bulb disrupts the ionic balance of plants and ultimately reduces bulb yield under salt stress (Zhou et al., 2021). The accumulation of biochemical changes like osmotic adjustment, antioxidant activity, ionic homeostasis, and proline content depicted a variable response of salt-tolerant and salt-sensitive genotype towards salinity stress (Beinșan et al., 2018**;** Mohammed Shamim Ahsan et al., 2022; Awad-Allah et al., 2020). Earlier reports suggested that the presence of a substantial amount of genetic variability for salinity tolerance in garlic genotypes makes the screening under salinity stress crucial for the selection of superior cultivars (Kovačević et al., 2023). The present study thus identified that salinity-tolerant genotypes and portioning of physiological, biochemical, ionic, and bulb yield traits contributed significantly for their use in future garlic breeding programs.

Based on percent reduction in bulb yield, genotypes were classified as salt-tolerant (≤15% yield reduction) and salt-sensitive (>30% yield reduction) groups (**Table 3**). Salt-tolerant genotypes Yamuna Safed-8 (G-384), G-304, G-324, Yamuna Safed (G-1), and Yamuna Purple-10 (G-404) maintained higher bulb yields, while salt-sensitive genotypes PGS-208, Yamuna Safed-5 (G-89), Agrifound White (G-41), GG-2, and Selection-459 showed pronounced yield reduction exceeding 30%. When reduction in bulb yield mean under salinity stress of all genotypes (**Table 5**) and the salt-tolerant group (**Table 3**) was compared, it was found that bulb yield reduction was less than 15% in the salt-tolerant group, whereas it was more than 20% in the overall group. A similar trend has been earlier reported in *Allium* species, where tolerant genotypes sustained growth and yield under salinity stress (Munns and Tester, 2008**;** Nana et al., 2024). The physiological, biochemical, and ionic changes under stress conditions were studied further in six salt-tolerant and five salt-sensitive genotypes. Previous observations also indicated a significant reduction in growth and yield traits of salt-sensitive *Allium* species under saline irrigation, indicating the susceptibility of sensitive genotypes to stress stimulated by salinity stress (Parihar et al., 2014). Bulb dimensional traits indicate significant sensitivity to salinity stress, with ED considerably more affected than PD (Ashraf and Harris, 2013).

Salinity stress affected growth and yield attributes of all the genotypes with significant reduction in PH (22.04%), bulb yield per plant (20.06%), ED (8.94%), PD (12.51%), TSS (5.10%), and no. of cloves per plant (9.29%) (**Table 5**). Physiological traits were also affected, and a significant reduction in MSI (15.22%), RWC (6.45%), g_s_ (15.83%), and Pn (15.69%) was shown in the Results section, which indicated impaired membrane integrity and gas exchange. Disruption of photosynthetic machinery under saline conditions contributed to reduced photosynthesis in genotypes that are sensitive to salt stress, and experimental results are corroborated with findings from earlier research (Ashraf and Harris, 2013; Singh et al., 2020; Abdelrady et al., 2024; Kokebie et al., 2024). Furthermore, Stavridou et al. (2020) reported reduced photosynthesis due to metabolic constraints like impaired stomatal conductance (g_s_), ultimately affecting bulb yield (Stavridou et al., 2020). Although photosynthetic rate declined in all the genotypes under salinity stress in our study, tolerant lines (G-384, G-404, G-324, and G-304) maintained relatively higher Pn and g_s_ values and thus maintained gas exchange and carbon assimilation despite osmotic and ionic stress caused by salinity; a similar pattern of results was also reported by Chaves et al. (2009). In plants, maintaining a high value for RWC and MSI is a key indicator of high cellular turgidity and minimal cellular or membrane damage (Sanwal et al., 2022). Moreover, the Na^+^/K^+^ ratio also acts as a salinity stress indicator in plants. In the current study, an increased Na^+^/K^+^ ratio was observed in roots (141.77%), shoots (119.59%), and bulbs (55.17%). Salinity causes plants to accumulate higher levels of Na^+^ with comparatively less K^+^, which disturbs the Na^+^/K^+^ balance (Parvin et al., 2019; Sheoran et al., 2021). This excessive accumulation between Na^+^ and K^+^ has been reported to inhibit plant growth, causing poor bulb development due to osmotic stress and decreased water uptake and ion toxicity in garlic plants (Acosta-Motos et al., 2017; Zhu, 2001). An imbalanced Na^+^/K^+^ ratio also causes a reduction in photosynthesis by increasing stomatal sensitivity in salt marsh plant species (Arif et al., 2020). In addition to ionic imbalance, increased osmotic potential and salt stress impair key physiological processes, including cell expansion, stomatal development, and stomatal regulation (Zorb et al., 2019).

Proline accumulation is widely recognized as a key adaptive response in plants exposed to salinity stress. In the present investigation, higher proline levels observed in tolerant genotypes (G-282 and G-384) suggest enhanced osmotic adjustment and protection of cellular structures. Proline acts not only as an osmolyte but also as a stabilizer of proteins and membranes, thereby maintaining cellular integrity under adverse conditions (Kaur et al., 2021; Al-Jomah et al., 2024). This contrasts with sensitive genotypes, where limited proline accumulation reflects weaker stress adaptation mechanisms. Salt stress also induces oxidative damage, as evidenced by increased levels of H_2_O_2_ and MDA in sensitive genotypes such as S-459, PGS-208, and GG-2. These compounds serve as reliable indicators of oxidative stress, representing excessive production of ROS and membrane lipid peroxidation (Gill and Tuteja, 2010). Elevated ROS levels can disrupt cellular homeostasis, leading to impaired growth and productivity. To mitigate oxidative damage, plants activate an efficient antioxidative defense system. Enzymes such as CAT, SOD, APX, and POX play crucial roles in scavenging ROS. The significantly higher activity of CAT, SOD, and POX in tolerant genotypes like G-404 and G-282 indicates a stronger detoxification capacity under salt stress conditions. This enhanced enzymatic activity reduces oxidative damage and contributes to improved stress tolerance. Previous studies have also linked increased antioxidant enzyme activity with better yield stability in garlic under stress environments (Ashraf, 2009; Hassan and Ali, 2014; Shubham et al., 2025).

Stepwise regression performed separately in salinity and control condition revealed ED as the main determinant of garlic bulb yield. Under control conditions, the findings indicate that PD and PH inclusion significantly improved the model, accounting for 81.69% of yield variability. These findings tell us the importance of bulb size and overall plant vigor in determining bulb yield. Similar results have been reported by Astaneh et al. (2019) where bulb diameter, PH, and biomass accumulation were significantly associated with improved garlic growth and bulb yield under saline environments. Under salinity stress, refinement of models included physiological and ionic traits, which was not the case under control conditions. These results suggest that under salinity stress, yield determinants, such as plant vigor, and physiological, ionic, and osmotic balance play a crucial role.

## 5 Conclusion

The study confirms that higher-yielding garlic cultivars can sustain superior productivity even under salinity stress, despite experiencing some reduction. Genotypes G-304, G-384, G-1, G-282, G-324, and G-404 can be utilized as salt-tolerant cultivars due to their minimal yield reduction and superior adaptive performance under saline environments. Their resilience is associated with the maintenance of higher relative water content, proline accumulation, membrane stability, and efficient physiological processes such as photosynthesis, transpiration, and stomatal conductance. Additionally, enhanced antioxidant enzyme activities (CAT, APX, SOD, and POX) played a crucial role in mitigating oxidative damage under stress. In contrast, genotypes Yamuna Safed-4 (G-323), Yamuna Safed-5 (G-189), and G-363, although high yielding, were moderately sensitive to salinity. Stepwise regression analysis highlighted equatorial bulb diameter as the most significant determinant of yield under both normal and saline conditions, while ionic traits gained importance specifically under stress. Overall, salt-tolerant genotypes G-404, G-1, and G-282 can be effectively utilized both as high-performing cultivars and as valuable parental lines in breeding programs. Furthermore, traits such as bulb diameter and ionic balance serve as selection criteria for breeding programs aimed at improving garlic productivity in saline environments.

## Data Availability

The original contributions presented in the study are included in the article/**Supplementary Material**. Further inquiries can be directed to the corresponding author.
