# Supplementary material for: Genotypic variation in garlic (Allium sativum L.) for salinity tolerance: role of antioxidant enzymes and bulb ionic ratios in yield maintenance
Source: Front Plant Sci. 2026 May 12;17:1825262. doi: 10.3389/fpls.2026.1825262 (PMC13202381; doi:10.3389/fpls.2026.1825262)
Supplement: Supplementary file 1 [file Table1.docx]

**Table 1: Passport details of the tested genotypes**

| **S.No.** | **Names of Varieties**  **/Elite lines** | **Cultivar**  **/Line** | **Year of release** | **Maturity (days)** | **Bulb colour** | **Developer Institute/**  **Collection Site** |
| --- | --- | --- | --- | --- | --- | --- |
| **G01** | Selection-459 | Cultivar | - |  |  |  |
| **G02** | Agrifound White (G-41) | Cultivar | 1989 | 140-145 | White | NHRDF* |
| **G03** | Yamuna Safed-4 (G-323) | Cultivar | 2006 | 140-150 | White | NHRDF |
| **G04** | Yamuna Safed-2 (G50) | Cultivar | 1996 | 140-160 | White | NHRDF |
| **G05** | Yamuna Safed-5 (G-189) | Cultivar | 2012 | 150-160 | White | NHRDF |
| **G06** | Yamuna Safed-8 (G-384) | Cultivar | 2015 | 150-160 | White | NHRDF |
| **G07** | Bhima Purple | Cultivar | 2011 | 135-140 | Purple | ICAR-DOGR** |
| **G08** | Bhima Omkar | Cultivar | 2009 | 120-135 | white | ICAR-DOGR |
| **G09** | Yamuna Safed-9 (G-386) | Cultivar | 2016 | 150-160 | White | NHRDF |
| **G10** | Yamuna Purple-10 (G-404) | Cultivar | - | 165-175 | Purple | NHRDF |
| **G11** | Yamuna Safed (G-1) | Cultivar | 1991 | 140-150 | White | NHRDF |
| **G12** | Yamuna Safed-3 (G-282) | Cultivar | 1999 | 120-140 | White | NHRDF |
| **G13** | G-324 | Advance line |  | 150-160 | White | NHRDF |
| **G14** | G-304 | Advance line |  | 145-155 | White | NHRDF |
| **G15** | G-363 | Advance line |  | 150-155 | White | NHRDF |
| **G16** | G-378 | Advance line |  | 145-150 | White | NHRDF |
| **G17** | PGS-200 | Breeding line |  |  | White | ICAR-IARI*** |
| **G18** | PGS-201 | Breeding line |  |  | White | ICAR-IARI |
| **G19** | PGS-202 | Breeding line |  |  | White | ICAR-IARI |
| **G20** | PGS-203 | Breeding line |  |  | White | ICAR-IARI |
| **G21** | PGS-204 | Breeding line |  |  | White | ICAR-IARI |
| **G22** | PGS-205 | Breeding line |  |  | White | ICAR-IARI |
| **G23** | PGS-206 | Breeding line |  |  | White | ICAR-IARI |
| **G24** | PGS-207 | Breeding line |  |  | White | ICAR-IARI |
| **G25** | PGS-208 | Breeding line |  |  | Purple | ICAR-IARI |
| **G26** | PGS-209 | Breeding line |  |  | White | ICAR-IARI |
| **G27** | PGS-210 | Breeding line |  |  | White | ICAR-IARI |
| **G28** | PGS-211 | Breeding line |  |  | White | ICAR-IARI |
| **G29** | PGS-212 | Breeding line |  |  | White | ICAR-IARI |
| **G30** | PGS-215 | Breeding line |  |  | White | ICAR-IARI |
| **G31** | PGS-216 | Breeding line |  |  | White | ICAR-IARI |
| **G32** | PGS-217 | Breeding line |  |  | White | ICAR-IARI |
| **G33** | GG-2 | Cultivar | 2000 | 135-140 | White | JAU, Junagarh |
| **G34** | GG-4 | Cultivar | 2009 | 130-140 | White | JAU, Junagarh |
| **G35** | Godawari | Cultivar |  | 140-145 | Purple | MPKV, Rahuri |
| **G36** | Phule Baswant | Cultivar | 2007 | 135-140 | Purple | MPKV, Rahuri |

*NHRDF: National Horticultural Research and Development Foundation, New, Delhi

**ICAR-DOGR: Directorate of onion and garlic research, Nasik, India

***ICAR-IARI: Indian Agricultural Research Institute, New Delhi, India
